# MND1 enables homologous recombination in somatic cells primarily outside the context of replication

**DOI:** 10.1002/1878-0261.13448

**Published:** 2023-06-14

**Authors:** Lisa Koob, Anoek Friskes, Louise van Bergen, Femke M. Feringa, Bram van den Broek, Emma S. Koeleman, Ellis van Beek, Michael Schubert, Vincent A. Blomen, Thijn R. Brummelkamp, Lenno Krenning, René H. Medema

**Affiliations:** ^1^ Division of Cell Biology, Oncode Institute The Netherlands Cancer Institute Amsterdam The Netherlands; ^2^ Bioimaging Facility The Netherlands Cancer Institute Amsterdam The Netherlands; ^3^ Division of Biochemistry, Oncode Institute The Netherlands Cancer Institute Amsterdam The Netherlands; ^4^ Present address: Department of Functional Genomics, Center for Neurogenomics and Cognitive Research (CNCR) VU University Amsterdam 1081 Amsterdam The Netherlands; ^5^ Present address: Chromatin Networks German Cancer Research Center (DKFZ) 69120 Heidelberg Germany; ^6^ Present address: Scenic Biotech 1098 XG Amsterdam The Netherlands; ^7^ Present address: Crown Bioscience 3584 CM Utrecht The Netherlands

**Keywords:** DNA damage response, double‐strand breaks, homologous recombination, irradiation

## Abstract

Faithful and timely repair of DNA double‐strand breaks (DSBs) is fundamental for the maintenance of genomic integrity. Here, we demonstrate that the meiotic recombination co‐factor MND1 facilitates the repair of DSBs in somatic cells. We show that MND1 localizes to DSBs, where it stimulates DNA repair through homologous recombination (HR). Importantly, MND1 is not involved in the response to replication‐associated DSBs, implying that it is dispensable for HR‐mediated repair of one‐ended DSBs. Instead, we find that MND1 specifically plays a role in the response to two‐ended DSBs that are induced by irradiation (IR) or various chemotherapeutic drugs. Surprisingly, we find that MND1 is specifically active in G2 phase, whereas it only marginally affects repair during S phase. MND1 localization to DSBs is dependent on resection of the DNA ends and seemingly occurs through direct binding of MND1 to RAD51‐coated ssDNA. Importantly, the lack of MND1‐driven HR repair directly potentiates the toxicity of IR‐induced damage, which could open new possibilities for therapeutic intervention, specifically in HR‐proficient tumors.

## Graphical abstract

Hypothetical model of the specific action of MND1/HOP2 complex on the repair of DSBs. We observe that the meiotic recombination co‐factor MND1 facilitates the repair of DSBs in somatic cells. Surprisingly, we find that MND1 is specifically active in G2 phase, whereas it only marginally affects repair during S phase.

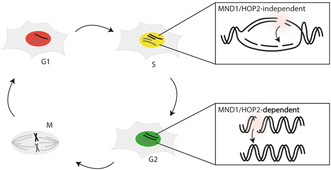

**Abbreviations**
ALT: alternative lengthening of telomeres
Co‐IP: co‐immunoprecipitation
DDR: DNA damage response
DSBs: Double‐strand breaks
HR: homologous recombination
ICL: intrastrand DNA crosslinks
IR: irradiation
MMEJ: microhomology‐mediated end‐joining
NHEJ: nonhomologous end‐joining
PARPi: PARP inhibitors
SSA: single‐strand annealing
ssDNA: single‐stranded DNA
UV: ultraviolet

## 1 Introduction

The integrity of the human genome is constantly challenged by a wide variety of endogenous and exogenous DNA damaging sources. To resolve DNA breaks, cells have evolved an intricate network of proteins that sense and repair the damage, collectively known as the DNA damage response (DDR) [1, 2, 3]. A dysfunctional DDR has been shown to drive cancer progression and evolution by accelerating the accumulation of mutations [4, 5]. Hence, mutations in DDR genes are commonly found as driver mutations in cancer. Such mutations often lead to inactivation of specific repair pathways and, as a consequence, cancers driven by mutations in DDR genes are often hypersensitive to loss or chemical inhibition of the alternative repair pathways [6, 7, 8]. This indicates the importance of a careful delineation of distinct repair pathways, as their redundancies create the potential for novel anticancer therapies.

A widely effective DNA damage‐inducing cancer treatment in the clinic is radiotherapy [9]. The detrimental effect of radiotherapy is mainly based on the induction of DNA double‐strand breaks (DSBs) by ionizing radiation (y‐irradiation, IR). DSBs are one of the most deleterious types of DNA damage [10, 11]. DSBs are repaired by a plethora of different repair pathways: non‐homologous end‐joining (NHEJ), homologous recombination (HR), single‐strand annealing (SSA), or microhomology‐mediated end‐joining (MMEJ). While NHEJ functions throughout the cell cycle, HR and SSA are mainly restricted to S and G2 phases [12], whereas MMEJ requires passage through mitosis [13, 14]. This is in part because HR is dependent on a homologous DNA strand, which is only present post replication during S phase. For HR to occur, DNA resection is required to generate a template for strand invasion. In contrast, NHEJ only requires minimal DNA end processing before ligation of broken ends [15, 16]. Resection generates stretches of single‐stranded DNA (ssDNA) that are coated by RPA. Later, RPA is exchanged for the RAD51 recombinase, which enables homology search and strand invasion of the sister chromatid [17, 18]. RAD51‐mediated homology search and strand invasion is facilitated by its ATP binding, whose inhibition abolishes DNA strand exchange and successful HR repair [19, 20].

Although much is known about the DDR network, genome‐wide screening in the context of various DNA damaging treatments continues to identify genes that are involved in DNA repair. This is highlighted by the recent discovery of DDR factors like Shieldin, ELOF1, and ERCC6L2 [21, 22, 23, 24]. Further identification of DDR genes will generate a more complete picture of the distinct repair pathways, potentially leading to the identification of novel (adjuvant) therapeutic targets. Therefore, we set out to identify factors that limit the toxicity of DSBs that are induced by IR. For this, we performed a haploid genetic screen aimed to identify genes involved in the cellular survival in response to IR. The screen identified many genes with well‐established roles in the DNA damage response, as well as several genes with no previously described role in DNA repair.

One of the most prominent hits in our screen was MND1, loss of which resulted in a marked increase in sensitivity to IR. This finding was surprising as MND1 is known for its role in homology search and recombination during meiosis, but not in somatic cells. Specifically, loss of MND1 has been shown to cause the persistence of meiotic DSBs and results in the formation of nonhomologous synapses [25]. During meiotic recombination, MND1 binds its co‐factor HOP2 and stabilizes both RAD51‐ and DMC1‐coated presynaptic filaments, which facilitates strand invasion and D‐loop formation [26, 27, 28]. MND1 has not been directly implicated in DSB repair during the somatic cell cycle. However, MND1 has been shown to facilitate the alternative lengthening of telomeres (ALT) [29], a process of telomere replication that is dependent on many established HR factors.

Here, we describe that MND1 facilitates DSB repair through HR also during the somatic cell cycle. We find that loss of MND1‐HOP2 complex sensitizes cells to DSBs induced by IR and various chemotherapeutic drugs. Interestingly, we find that MND1 is dispensable for HR‐dependent repair of replication‐associated breaks, indicating that targeting MND1 can be a way to inhibit some, but not all, HR‐dependent repair. MND1 localizes readily to DSBs where it facilitates the timely resolution of RAD51 foci and stimulates HR. Consequently, MND1 loss potentiates the G2 DNA damage checkpoint, causing hypersensitivity to DNA damage during G2 phase. Therefore, we conclude that MND1 has a critical role in the repair of DSBs via HR during the somatic cell cycle.

## 2 Materials and methods

### 2.1 Cell culture

Human‐derived near‐haploid HAP1 (RRID: CVCL_Y019, Brummelkamp Laboratory, Amsterdam, The Netherlands) cells were cultured in IMDM (GIBCO, Waltham, USA) supplemented with 12% FCS, 1% GlutaMAX (GIBCO, Waltham, USA) and 100 U·mL^−1^ penicillin–streptomycin. RPE1 (RRID: CVCL_4388, ATCC, Manassas, USA; CCNB1‐YFP, Fucci and ΔP53), HCT116 (RRID: CVCL_0291), and SAOS2 (CVCL_0548) cells were cultured in DMEM:F12 (GIBCO, Waltham, USA) supplemented with 6% FCS, 1% GlutaMAX (GIBCO, Waltham, USA), and 100 U·mL^−1^ penicillin–streptomycin. U2OS (RRID: CVCL_0042) DR‐GFP/ SA‐GFP (Stark Laboratory [30]) and Fucci cells were cultured in DMEM (GIBCO, Waltham, USA) supplemented with 6% FCS, 1% GlutaMAX (GIBCO, Waltham, USA), and 100 U·mL^−1^ penicillin–streptomycin. H1299 (RRID: CVCL_0060, ATCC) cells were cultured in RPMI 1640 (GIBCO) supplemented with 12% FCS, 1% GlutaMAX (GIBCO), 1% HEPES (GIBCO, Waltham, USA), 1% MEM nonessential amino acids (GIBCO, Waltham, USA), and 100 U·mL^−1^ penicillin–streptomycin.

All cell lines were tested negatively for mycoplasm before experiments were performed and authenticated prior to use (ANSI/ATCC standard ASN‐0002, using the Applied Biosystems™ AmpFLSTR™ Identifiler™ Plus PCR Amplification Kit system, performed by Eurofins Genomics, Luxemburg). Of note: the used HCT116 cell line was identified to overlap with the certified HCT116 (RRID: CVCL_0291) with ~ 75%. This is possibly due to ongoing mutagenesis caused by MSI.

### 2.2 Haploid genetic screen

Genes essential for the fitness of cells treated with y‐irradiation were identified as previously described [31]. In brief, gene‐trap retrovirus was produced in HEK293T cells. After harvesting the virus, approximately 40 million HAP1 cells were mutagenized. The mutagenized cells were treated with y‐irradiation (1 Gy, every other day) and passaged for 10 days in total. After passaging, cells were collected and fixed. Fixed cells were stained with DAPI to allow sorting for haploid cells only. The genomic DNA was isolated using a DNA mini kit (QIAGEN, Venlo, The Netherlands). The gene‐trap insertion sites were amplified by LAM‐PCR and sequenced using primers containing Illumina adapters [31]. Mapping and analysis of insertion sites is described in detail [32]. In short, sequence reads were aligned to the human genome (hg19) to obtain the genomic locations of insertion sites. Subsequently, the gene‐trap insertions were intersected with Refseq gene coordinates to ascertain intragenic integrations and the orientation with respect to the transcriptional direction of the gene. Overlapping gene regions that introduce ambiguity to insertion site direction calling were disregarded. To identify genes that are affecting fitness in IR‐treated cells, the sense and antisense orientation integrations for each were compared with those in four independent published untreated datasets [31]; NCBI SRA accession no. SRP058962) using a Fisher's exact test.

### 2.3 Ionizing radiation and clonogenic outgrowth

Cells were irradiated using a Gammacell Exactor (Best Theratronics, Ottawa, Canada) with a ^137^Cs source. For assessing the sensitivity of cell lines toward y‐irradiation, low amounts of cells were plated per well, treated with different doses of irradiation, and grown into single colonies for 6 days. The number of colonies was then counted and normalized to the untreated condition.

### 2.4 GO term enrichment analysis

Hits from the haploid genetic screen were analyzed for gene ontology (GO) enrichment using http://geneontology.org/ (release 2022‐07‐01: 43.558) [33, 34].

### 2.5 siRNA and crRNA transfections

siRNA transfections were performed using RNAiMAX (Invitrogen, Waltham, USA) according to the manufacturer's guidelines. The following siRNAs were used in this study: siNT (nontargeting; Dharmacon, Cambridge, UK), siMND1 (Dharmacon, Cambridge, UK), 3′UTR siMND1 (5′‐UUCTUUGTGTUCTGUUTTGTCG‐3′) siPSMC3IP (HOP2; Dharmacon, Cambridge, UK), siRAD51 (Dharmacon, Cambridge, UK), siBRCA1 (Dharmacon, Cambridge, UK), siBRCA2 (Dharmacon, Cambridge, UK), siPRKDC (DNAPKc; Dharmacon, Cambridge, UK), and siTP53 (p53; Dharmacon, Cambridge, UK).

A crRNA targeting exon 4 of MND1 (ΔMND1 cell line generation, 5′‐CAAGTAAAGCTCTTCATGCA‐3′), exon 2 of HOP2 (ΔHOP2 cell line generation, 5′‐CCGGGATCCTCCTGAGGTAC‐3′), GAPDH pseudogenes (P63, 5′‐AACGGGAAGCTTGTCATCAA‐3′) [35], or telomeres (Telo, 5′‐UUAGGGUUAGGGUUAGGGUU‐3′) [36] were transfected using RNAiMAX (Invitrogen, Waltham, USA). In brief, 20 nm crRNA and tracrRNA are incubated with RNAiMAX (Invitrogen, Waltham, USA) in OptiMEM (GIBCO, Waltham, USA). After 20 min of incubation, the transfection mix is added to the cells. Indel generation in the MND1 locus is validated by TIDE [37] analysis of PCR products.

### 2.6 CRISPRi‐mediated knockdown

Two MND1 sgRNAs (#1 5′‐GCGGCGAAGCCCACACACTA‐3′, #2 5′‐GCTGCGCCCGCGCCATGGTA‐3′) targeting the promoter were cloned into a pLV‐sgRNA plasmid and lentivirally transduced into cells.

### 2.7 Generation of (GFP‐)MND1 overexpression cell lines

pCW_MND1 plasmid was cloned by PCR amplification of MND1 from cDNA using the following primers:

fwd 5′‐TGAACCGTCAGATCGCCTGGAGAATTGGTCGACatgtcaaagaaaaaaggactgagtgca‐3′ rev 5′‐AAAAGGCGCAACCCCAACCCCACGCGTttagtctatgtagtcaaagtcttctggaattcc‐3′.

pCW_GFP‐MND1 plasmid was cloned by PCR amplification of both GFP and MND1 from plasmid DNA or cDNA with the following primers:

fwd1 5′‐TGAACCGTCAGATCGCCTGGAGAATTGACCGGTATGAGCAAAGGAGAAGAACTTTTCACT‐3′

rev1 5′‐tgcactcagtccttttttctttgaACCACTTCCTCCAGCCGAAGCGCTTTTGTAGAGC‐3′.

fwd2 5′‐GCTCTACAAAAGCGCTTCGGCTGGAGGAAGTGGTtcaaagaaaaaaggactgagtgca‐3′.

rev2 5′‐AAAAGGCGCAACCCCAACCCCACGCGTttagtctatgtagtcaaagtcttctggaattcc‐3′.

The PCR products were ligated into a pCW vector with BFP‐T2A‐Blast/Puro selection marker by Gibson ligation.

Plasmids were then lentivirally transduced into HAP1 and RPE1 cells, selected for blast selection, and sorted for BFP+.

### 2.8 Clonogenic outgrowth assay/drug‐response assays

To assess colony outgrowth after irradiation, 250 cells were seeded per well in 6‐well plates. Cells were fixed after 7 days of growth in 80% methanol and stained with 0.2% crystal violet. Colonies were counted and normalized to the unirradiated control.

For drug‐response assays, 500 cells were plated per well in 96‐well plates. After treatment with various drugs, cells were grown for 7 days. Cells were fixed in 80% methanol and stained with 0.2% crystal violet. Crystal violet staining was analyzed after treatment with 10% acetic acid in water. Intensity of staining was then quantified using a Biotek Epoch Microplate Reader.

### 2.9 Endogenously tagging of 53BP1 and RPA1

CRISPR/Cas9 was used to create endogenously tagged RPE1 iCut cell lines with HALO‐53BP1 and RPA1‐mScarlet2. For tagging endogenous 53BP1 N‐terminally with HALO, a 3x‐HA‐HALO‐tag flanked with 200 bp homology arms was acquired in a gBlock. RPA1 was tagged at the C‐terminal site with the mScarlet2 fluorophore, a mScarlet2‐3x‐HA flanked with 90 bp homology arms was acquired in a gBlock. A mutation in the PAM sequence was introduced to prevent re‐cutting by pSpCas9 after initial break repair. The gBlocks were cloned into a KpnI‐XbaI digested pUC19 vector (Addgene, Watertown, USA, #50005) by Gibson assembly. To insert the tags, CRISPR/Cas9‐induced DSBs were generated using the pSpCas9(BB)‐2A‐Puro (PX459) plasmid (Addgene, Watertown, USA, #62988) expressing the following sgRNAs that were designed in close proximity to or overlapping with the start codon of 53BP1 and the stop codon of RPA1 using crispor and blast: 53BP1 5′‐GAGCGCGAGGGACCTCCCGCC‐3′ and RPA1 5′‐GAGAAGTGCATTGATGTGAG‐3′. For nucleofection, RPE‐1 iCut cells were plated and treated for 48 h with p53 siRNA (see siRNA transfections), M3814 (MCE, 1 mm), doxycycline (Sigma, St. Louis, USA, 1 mm), and SHIELD‐1 (Aobious, Gloucester, USA, 1 μm). Nucleofection was performed using Amaxa Nucleofector treating cells with 1 μg pUC19 plasmid, 1 μg PX459 plasmid and blasticidin‐expressing plasmid in Amaxa buffer (Lonza, Basel, Switzerland) with 100 mm ATP. After nucleofection, cells were plated and selected with blasticidin (10 μg·mL^−1^) for 48 h. To generate monoclonal endogenously tagged cell lines, successfully nucleofected cells were identified by FACS (BD FACSAria, BD biosciences, Franklin Lakes, USA). HALO‐53BP1 cells were incubated with a HALO‐ligand, following the ligand labeling protocol. RPA1‐mScarlet2 cells were identified by red fluorescence. Single clones were grown out and tested for endogenous knock‐in by performing PCRs outside homology arms: OH‐ARM_53BP1 FWD 5′‐TCCATGCTGCCATGGAAACG‐3′ and REV 5′‐AATCTGTTCGCCAGAGGCCC‐3′, HALO‐FWD 5′‐ATGGCAGAAATCGGTACTGG‐3′.

### 2.10 Immunofluorescence staining and fixed‐cell imaging

Cells were pre‐extracted using 0.5% Triton X‐100 in PBS on ice for 30 s and immediately fixed on coverslips for 15 min at room temperature (RT) using a final concentration of 3.5% formaldehyde. Then, cells were permeabilized for 5 min using 0.5% Triton X‐100 in PBS. Cells were blocked in PBS supplemented with 0.1% Tween‐20 (PBS‐T) with 5% bovine serum albumin (BSA) for 1 h. Primary antibody incubation was performed at RT for 1.5 h (antibodies and dilutions stated below). Coverslips are washed with BSA in PBS‐T, and secondary antibody incubation is performed at RT for 1 h. After incubation with secondary antibody, coverslips were washed with PBS. EdU was stained by incubation in EdU staining buffer (100 mm Tris–HCl pH 8.5, 1 mm CuSO_4_), with 100 mm ascorbic acid and AF‐647 azide (Invitrogen, Waltham, USA, 1/1000) for 30 min at RT. After washing three times with PBS‐T, coverslips were mounted on microscopic slides using Prolong Gold (Invitrogen, Waltham, USA) and stored at 4 °C.

### 2.11 Immunofluorescence and live‐cell imaging

Cells were either fixed and stained as described above or grown in Lab‐Tek II chambered coverglass (Thermo Scientific, Waltham, USA) in tissue culture medium outfitted with a CO_2_ controller set at 5%. Images for Fig. 2 were obtained using a DeltaVision Elite (Applied Precision, Bratislava, Slovakia) maintained at 37 °C and 5% CO_2_ equipped with a 40× and 63× PLANApo S lens (Olympus, Tokyo, Japan) and cooled CoolSnap CCD camera. Images for Fig. 4 were obtained using a THUNDER Imager (Leica Microsystems, Wetzlar, Germany) maintained at 37 °C and 5% CO_2_ equipped with a 63×/1.40–0.60 OIL Obj. HC PL APO objective and a deep‐cooled 4.2 MP sCMOS camera.

### 2.12 Foci quantification

For foci quantification in Fig. 2, a previously published ImageJ macro was used [38].

For foci quantification in Fig. 4, images were split into single timepoints. Nuclear foci were quantified in fiji [39], using a custom‐built ImageJ macro that enabled automatic and objective foci analysis https://github.com/BioImaging‐NKI/Foci‐analyzer. Initially, cell nuclei were detected by thresholding the (median‐filtered) DAPI signal, followed by a watershed operation to separate touching nuclei. In a later version, stardist [40] was used for nuclei segmentation.

Brief outline of the foci detection workflow (in 2D): After maximum intensity z‐projection, the foci signal is background‐subtracted using a Difference‐of‐Gaussians filter. Foci candidates are identified as local maxima exceeding a user‐adjustable threshold. These maxima are then used as seeds for MorpholibJ's [41] marker‐controlled watershed segmentation, executed on the gpu using clij2/clijx [42], followed by size filtering to exclude very small foci. Overlay images of segmented nuclei, detected foci and original signals provide a convenient way to inspect the results and optimize parameters, depending on foci size, intensity and noise levels. In experiments with two foci channels, foci are considered co‐localized if their spatial overlap is at least 1 pixel.

### 2.13 Western blot

Western blot analysis was performed as described previously [38]. In brief, proteins were separated using SDS‐polyacrylamide gel electrophoresis and transferred to nitrocellulose membranes. Membranes were blocked in 5% BSA in PBS‐T and afterward incubated with primary antibodies overnight at 4 °C (dilutions of antibodies indicated below). Secondary antibodies are incubated as stated below for 1 h at room temperature. Proteins were visualized using enhanced chemiluminescence (ECL; GE Healthcare, Chicago, USA).

### 2.14 Antibodies and chemicals

The following primary antibodies were used in this study: anti‐H2AX ser139 (yH2AX, 05‐636, Upstate, Burlington, USA, 1 : 500 IF, 1 : 1000 WB), anti‐RAD51 (ab63801, Abcam, Cambridge, UK, 1 : 500 IF), anti‐pCHK1 ser345 (2344, Cell Signaling, Danvers, USA, 1 : 500 WB), anti‐pCHK2 thr68 (2661, Cell Signaling, Danvers, USA, 1 : 1000 WB), anti‐GFP (11814460001, Roche, Basel, Switzerland, 1 : 1000 WB/IF), anti‐HA (H9658, Sigma, Waltham, USA, 1 : 1000 WB), anti‐histone H3 (ab1791, Abcam, Cambridge, UK, 1 : 1000 WB), anti‐HSP90 (sc7947, Santa Cruz, Dallas, USA, 1 : 1000 WB), anti‐MND1 (235395, Abcam, Cambridge, UK, 1 : 1000 WB), anti‐HOP2 (11339‐1‐ap, Thermo, Waltham, USA, 1 : 1000 WB), and anti‐pATM ser1981 (#4526, Cell Signaling, Danvers, USA, 1 : 1000 WB).

The following secondary antibodies were used for western blot experiments: peroxidase‐conjugated goat anti‐rabbit (P448, DAKO, Santa Clara, USA, 1 : 1000) and goat anti‐mouse (P0447, DAKO, Santa Clara, USA, 1 : 1000). Secondary antibodies used for immunofluorescence were goat anti‐mouse‐Alexa 488 (A11029, Mol Probes, Waltham, USA, 1 : 1000) and goat anti‐rabbit‐Alexa 568 (A11011, Mol Probes, Waltham, USA, 1 : 1000).

Chemicals used in this study: DNAPK inhibitor (NU‐7441; 14881, Cayman, Ann Arbor, USA), olaparib (AZD2281, M1664, Bioconnect, Huissen, The Netherlands), talazoparib (AZD2461, SML1858, Sigma, Waltham, USA), RAD51 inhibitor (B02, 553 525, Merck, Burlington, USA), neocarzinostatin (NCS, N9162, Sigma, Waltham, USA), doxorubicin (D1515, Sigma, Waltham, USA), etoposide (E1383, Sigma, Waltham, USA), zeocin (R250‐01, Invitrogen, Waltham, USA), mitomycin c (MMC, M0503, Sigma, Waltham, USA), cisplatin (P4394, Sigma, Waltham, USA), hydroxyurea (HU, 0210202310, MP Biomedicals, Santa Ana, USA), aphidicolin (A0781, Sigma, Waltham, USA), and camptothecin (CPT, 0215973225, MP Biomedicals, Santa Ana, USA).

### 2.15 Flow cytometry analysis and sorting

Cells were trypsinized and resuspended in PBS supplemented with 1% FCS for sorting, using a BD FACSAria Fusion. G2 cells were sorted based on Cyclin B1‐YFP signal and replated for colony counting or harvested for propidium iodide (PI) staining. RPE1 Fucci cells were sorted based on Azami‐Green and Kusabira‐Orange signal depending on cell cycle phase (gating strategy indicated in Fig. S3C) and replated for colony counting.

GFP^+^ and BFP^+^ cells were analyzed using BD LSRFortessa after cells were harvested in trypsinization and resuspended in PBS + 1% FCS. Cell cycle distribution was analyzed by staining fixed cells with PI.

### 2.16 DR‐GFP assay

U2OS DR‐GFP cells were gifted by the Stark Laboratory, and the assay was performed as described in [30]. In brief, 72 h after siRNA transfection (see above), media were changed to antibiotics‐free DMEM media, and U2OS DR‐GFP cells were then transfected with 1 μg ISceI‐RFP expressing plasmid using Lipofectamine 2000. Three‐hours post‐transfection, media were changed and supplemented with triamcinolone acetonide (TA) to induce nuclear translocation of the ISceI protein. After 72 h, cells were harvested and RFP and GFP positivity were measured using a BD LSRFortessa.

## 3 Results

### 3.1 A haploid genetic screen identifies MND1 loss as sensitizing toward y‐irradiation

**Fig. 1:**
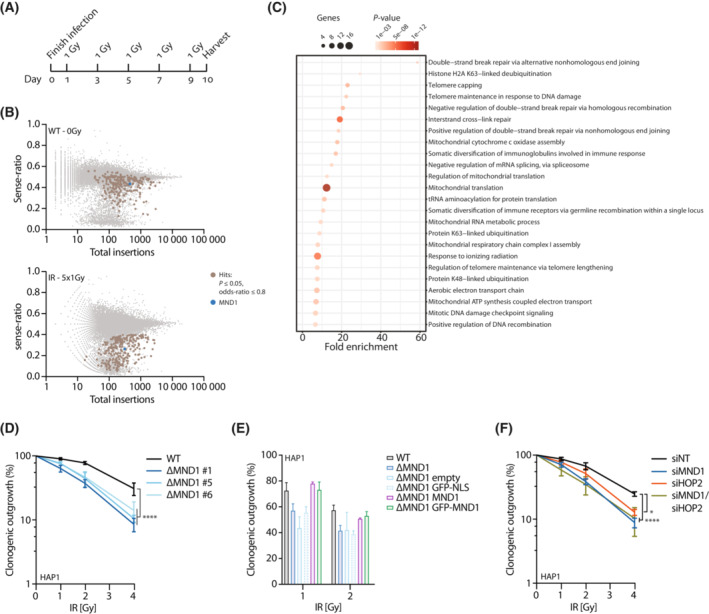
Haploid genetic screen identifies MND1 as a sensitizer to IR‐induced DNA damage. (A) Treatment schedule of the haploid genetic screen. Cells were treated with 1 Gy of IR every other day, five times. The cells were harvested 10 days after the start of the screen. (B) Results from the HAP1 genetic screen (Control—0 Gy (one dataset is displayed) and IR—5 × 1 Gy) are shown by plotting the relative amount of mapped sense integrations over total insertions. Hits are defined with *P*‐values ≤ 0.05 when compared against all four WT datasets and an odds ratio ≤ 0.8. These hits are highlighted in brown, and MND1 is highlighted in blue. (C) Gene ontology enrichment of screen hits from this study. (D) Clonogenic outgrowth of HAP1 WT and three monoclonal ΔMND1 cell lines (blue) in response to increasing doses of IR. Data presented as mean ± SEM, *N* = 3. (E) Clonogenic outgrowth of IR‐treated HAP1 WT and ΔMND1 cells overexpressing either doxycycline (Dox)‐inducible control plasmids (pCW_empty or pCW_GFP‐NLS) or pCW_(GFP‐)MND1 construct. Data presented as mean ± SEM, *N* = 3. (F) Colony outgrowth of HAP1 cells treated with siRNAs targeting MND1 and HOP2 in response to increasing doses of IR, nontargeting (NT) siRNA as control. Data presented as mean ± SEM, *N* = 3. (D–F) Statistics depict *P*‐values (*P ≤ 0.05, ****P ≤ 0.0001) after analysis using a Fisher's test.

To identify novel factors involved in the DNA damage response (DDR), we performed a genetic perturbation screen using random mutagenesis by gene‐trap insertion in haploid human cells (HAP1 cells) [31]. Gene function is mainly disrupted upon integration of the gene trap in the sense orientation. As such, gene essentiality is determined by calculating the ratio of sense versus antisense integrations. In this study, mutagenized cells were treated with 1 Gy IR five times every other day and compared with untreated control (WT–0 Gy) cell populations [31] (Fig. 1A, one representative WT dataset is depicted). After 10 days of culture, cells were collected and gene‐trap insertions were determined by next‐generation sequencing (see Methods for a detailed description of the screen setup). We defined a gene as a hit when it was enriched for antisense orientation gene‐trap insertions in the irradiated cell population (odds ratio ≤ 0.8), and the insertion ratio was significantly different compared with the four independent control datasets (*P*‐value ≤ 0.05) [31]. This resulted in a list of 261 genes (Table S1, highlighted in brown in Fig. 1B). Gene set enrichment analysis of the screen hits identified multiple pathways involved in the DNA damage recognition and repair to be significantly enriched (Fig. 1C), indicating that our screen setup is able to identify DDR factors.

To narrow down our hit list, we cross‐referenced our screen with both replicates of a recently published IR screen in HAP1 cells [23]. We applied the same cutoffs for all three IR datasets (*P*‐value ≤ 0.05 and an odds ratio ≤ 0.8), which identified 37 common hits (Fig. S1A,B). This list includes genes known for their role in the response to IR like the Shieldin complex [21, 22, 43], *PRKDC* (DNAPKc; [3, 44]), and *RNF168* [45], demonstrating that the screens were able to identify genes involved in the response to DNA damage. We also find several other genes including *CTDSPL2* (rank 9), *MND1* (rank 21), *RPRD2* (rank 23), and *PKM* (rank 36) with no previously described role in DNA repair in somatic cells. We were particularly interested that we identified *MND1* (highlighted in blue, Fig. 1B), which is known as a meiotic recombination factor, but for which a general role in the DNA damage response in somatic cells has yet to be described.

MND1 is well known for its role during meiotic crossover repair after DSB induction by the SPO11 nuclease [46, 47]. Interestingly, analysis of MND1 mRNA transcript levels shows expression in all analyzed tissue types to similar levels of RAD51 (Fig. S1C). This global expression across different tissue types is also observed for HOP2, the co‐factor of MND1. Therefore, we conclude that MND1‐HOP2 are ubiquitously expressed proteins. To address whether MND1 is involved in DNA repair in somatic cells, we first confirmed that loss of MND1 increases IR sensitivity in three independent HAP1 knockout clones (ΔMND1; Fig. 1D and Fig. S1D). Similarly, depletion of MND1 using either CRISPRi or siRNAs also causes increased sensitivity toward IR (Fig. S1E). We confirmed that the observed sensitivity toward IR in ΔMND1 cells is a direct result of the loss of MND1, as exogenous expression of either MND1 or GFP‐MND1 reduces IR sensitivity (Fig. 1E and Fig. S1F). In summary, our haploid genetic screen identified MND1, which we here establish to have an important role in the response to IR in somatic cells.

In meiotic cells, MND1 is bound to a co‐factor, HOP2 [48, 49]. This MND1‐HOP2 interaction is essential for the role of MND1 in the repair of SPO11‐mediated DSBs during meiosis [50]. However, our screen did not identify HOP2 (gene name: *PSMC3IP*). Upon closer inspection, we found that gene‐trap sense integrations in the *HOP2/PSMC3IP* locus were in fact decreased upon IR, but were only found significantly different when compared to three out of the four control datasets (Table S1). When we depleted HOP2 using siRNAs, we indeed found a significant sensitization of HAP1 cells toward IR (Fig. 1F). Furthermore, when we co‐depleted MND1 together with HOP2, we found a similar sensitization as after MND1 depletion alone (Fig. 1F). Similarly, siRNA‐mediated depletion of HOP2 in HAP1 ΔMND1 cells also did not result in any increased sensitivity toward IR (Fig. S1H). This indicates that MND1 and HOP2 indeed act together in the response to IR in HAP1 cells. As MND1 and HOP2 were shown to interact together during meiosis [48, 49, 51], we investigated whether they also interact in somatic cells. Co‐immunoprecipitation (Co‐IP) in cells expressing GFP‐MND1 or GFP alone demonstrated that MND1 and HOP2 indeed interact in somatic cells in a DNA damage‐independent manner (Fig. S1I). Together, these data confirm that MND1 and HOP2 act together in a complex during the response to IR in somatic cells, akin to their mutually dependent role during meiotic DNA damage repair.

After confirming that MND1 loss sensitizes HAP1 cells to IR, we aimed to confirm whether MND1 is important in the response to IR in different cell lines as well. The comparison of the LD50 of IR in control or MND1‐depleted cells demonstrates that MND1 loss sensitizes U2OS, H1299, RPE1ΔP53 and, to a limited extent, HCT116, but not SAOS2 cells (Fig. S1J). MND1 and HOP2 are expressed in all cell lines tested (Fig. S1K). Hence, the lack of MND1‐requirement in SAOS2 cells cannot be explained by differential expression of MND1 or HOP2. The only previously described role of MND1 in somatic cells is a role in ALT [29], a mechanism of telomere maintenance and extension that is closely related to HR [52, 53]. However, we do not find a correlation between the ALT status and IR sensitization (HAP1, RPE1ΔP53 and HCT116 cells are ALT^−^; H1299, U2OS, and SAOS2 cells are ALT^+^). Thus, ALT status can also not explain the lack of MND1‐requirement in SAOS2 cells. Furthermore, we have considered whether the cell cycle distribution of the cells can explain the differences we see between the cell lines. However, we find no correlation between the percentage of G2 phase cycling cells and the LD50 of IR (Fig. S1L). Collectively, we conclude that MND1 loss sensitizes most tested cell lines toward IR. Future work is necessary to understand the differential sensitivity of cell lines to the loss of MND1 in response to IR‐induced damage. This will shed more light on the dependencies of different genetic backgrounds to specific repair pathways.

### 3.2 MND1 facilitates DSB repair by assisting in homologous recombination

**Fig. 2:**
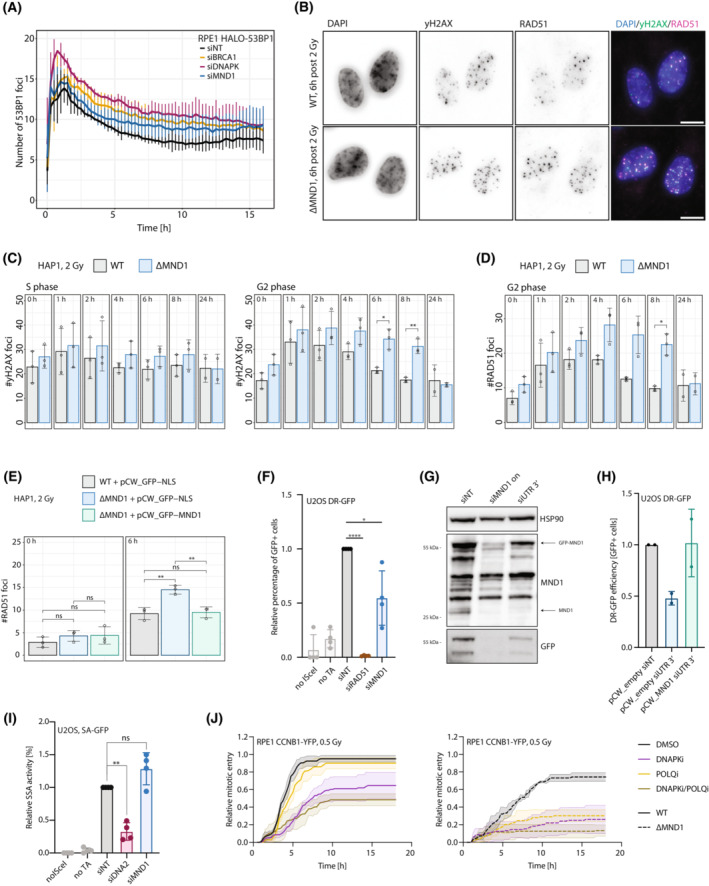
MND1 plays a role in the repair of DSBs via homologous recombination. (A) 53BP1 foci formation in RPE1 HALO‐53BP1_RPA1‐mScarlet2 cells after treatment with 4 Gy. Data presented as mean ± SD, *N* = 2. (B) Representative images of HAP1 WT and ΔMND1 cells stained for yH2AX and RAD51, *N* = 3. Scale bar: 10 μm. (C) Immunofluorescence staining of yH2AX foci in HAP1 WT and ΔMND1 cells during S and G2 phases of the cell cycle at different time points after IR with 2 Gy. Three independent replicates are displayed (mean ± SD), statistical analysis (*t*‐test) was performed on mean values (*P ≤ 0.05, **≤ 0.01). (D) Immunofluorescence staining of RAD51 foci in HAP1 WT and ΔMND1 G2 phase cells at different time points after IR with 2 Gy. Three independent replicates are displayed (mean ± SD), statistical analysis (*t*‐test) was performed on mean values (*P ≤ 0.05). (E) Immunofluorescence staining of RAD51 foci in HAP1 WT and ΔMND1 G2 phase cells expressing either pCW_GFP‐NLS or pCW_GFP‐MND1 plasmids either not irradiated or 6 h after 2 Gy. Three independent replicates are displayed (mean ± SD), statistical analysis (*t*‐test) was performed on mean values (**P ≤ 0.01). (F) HR frequency was assessed using the DR‐GFP assay in U2OS cells treated with siNT, siRAD51, and siMND1. DSBs were induced by transfection of ISceI nuclease plasmid and triamcinolone acetonide (TA) addition. Data presented as mean ± SD (*N* = 4). (G) Western blot analysis of GFP‐MND1 and endogenous MND1 expression in RPE1 cells after treatment with siNT, siMND1 (on‐target pool), or siMND1 3′. GFP protein expression was blotted as well as HSP90 levels as loading control (*N* = 2). (H) HR frequency was assessed using the DR‐GFP assay in U2OS cells expressing either pCW_empty or pCW_MND1, treated with siNT or siMND1 3′. DSBs were induced by transfection of ISceI nuclease plasmid and TA addition. Data presented as mean ± SD (*N* = 2). (I) SSA frequency was assessed using the SA‐GFP assay in U2OS cells expressing either pCW_empty or pCW_MND1, treated with siNT, siDNA2, or siMND1. DSBs were induced by transfection of ISceI nuclease plasmid and TA addition. Data presented as mean ± SD (*N* = 4). (J) RPE1 CCNB1‐YFP WT and ΔMND1 cells were treated with DMSO, DNAPKi, POLQi, or a combination before IR treatment with 0.5 Gy. Cells were followed from G2 phase into mitosis and time of mitotic entry analyzed (*N* = 3). (E, F, I) Statistics depict *P*‐values (*P ≤ 0.05, **P ≤ 0.01, ****P ≤ 0.0001) after analysis using an unpaired *t*‐test.

After establishing a role for MND1 in the response to IR, we wanted to identify the specific mode of action of MND1. During meiosis, repair of SPO11‐induced DSBs is critically dependent on the MND1‐HOP2 complex, and the retention of DNA damage in the absence of MND1‐HOP2 is well described [46, 48, 54]. To test whether DNA DSBs are also retained in somatic cells, we tested whether MND1 is involved in the repair of IR‐induced breaks in somatic cells. Hence, we first assessed DNA repair kinetics by quantification of 53BP1‐foci as a proxy for the appearance and subsequent repair of DNA DSBs. For this, we used live‐cell imaging of RPE1 cells in which we homozygously knocked in a HALO‐tag into the N‐terminal site of the 53BP1‐locus (Fig. S2A–E and see Methods for cell line generation). When analyzing the repair kinetics of 53BP1 foci in NT or MND1 siRNA‐treated cells, it is interesting to see that the initial rate of repair (until ~ 4 h) after IR is similar to NT cells after depletion of MND1. The MND1‐depleted cells however are impaired in the further resolution of 53BP1 foci and have a higher level of unresolved DNA damage at the end of the movie (until ~ 16 h after IR, Fig. 2A and Fig. S2F,G). This indicates that MND1 is involved in the repair of DSBs, but mainly for breaks that take a relatively long time to repair. When comparing the kinetics of 53BP1 foci resolution in MND1‐depleted cells to BRCA1‐ or DNAPKc‐depleted cells, we see a retention of DNA damage to a lesser extent. This indicates that MND1 has a less pronounced role in the DDR than BRCA1 or DNAPKc, which are known for their essential role in HR and NHEJ, respectively.

To explore whether MND1 acts at different stages during the somatic cell cycle, we assessed yH2AX focus formation and resolution in different cell cycle phases in HAP1 cells by immunofluorescence staining of yH2AX (representative images in Fig. 2B). Interestingly, loss of MND1 impaired yH2AX foci resolution only in G2 phase (Fig. 2C), suggesting that MND1 does not affect DNA repair in S phase. We confirmed that MND1 specifically affects DNA repair in G2 phase by analyzing 53BP1 foci formation and resolution (Fig. S2H). Collectively, these data demonstrate that MND1 is specifically involved in the repair of DSBs in G2 phase of the mitotic cell cycle.

Following SPO11‐induced break formation in meiosis I, the DSB is resected and MND1 acts to facilitate invasion of the single‐stranded section of the DSB into the intact double‐stranded homologous chromosome [47, 55, 56]. Given the established role of MND1 in meiotic recombination in germ cells, we next investigated whether MND1 is involved in HR in somatic cells. First, we assessed the sensitivity of MND1 knockout cells to different DSB repair pathway inhibitors. We found that the loss of MND1 increases the sensitivity specifically toward DNA‐PKcs inhibition, which inhibits DNA repair through NHEJ. Conversely, the sensitivity to inhibition of RAD51, and thus inhibition of HR, was unaltered in HAP1 ΔMND1 cells (Fig. S2I). The lack of sensitivity of ΔMND1 cells to RAD51 inhibitors implies that MND1 is redundant with the RAD51‐dependent HR repair. These data indicate that MND1 knockout cells rely on NHEJ, consistent with a (partial) deficiency in HR upon MND1 loss.

We next assessed at which step HR is compromised upon loss of MND1. To this end, we analyzed the appearance and disappearance of RAD51 foci after IR in the G2 phase. RAD51 is recruited to the DSB prior to strand invasion [57], and MND1 facilitates strand invasion in meiotic cells [28, 58]. We find that RAD51 recruitment occurs with similar kinetics in HAP1 ΔMND1 and WT cells (Fig. 2D). However, the resolution of RAD51 foci occurs markedly slower in ΔMND1 cells (Fig. 2D). Exogenous expression of GFP‐MND1 rescues this RAD51 repair defect (Fig. 2E). The observed repair defect is not HAP1‐specific as we see the same slower yH2AX and RAD51 resolution in RPE1 HALO‐53BP1 ΔMND1 and ΔHOP2 cells (Fig. S2J). These data indicate that MND1 knockout cells exhibit a partial defect in the completion of HR, possibly at the level of strand invasion. To confirm that MND1 facilitates HR repair during the somatic cell cycle, we used the DR‐GFP system to assess HR efficiency. In brief, induction of a DSB at a mutated, inactive, GFP can restore GFP fluorescence if that DSB undergoes HR repair [30]. MND1 depletion caused a reduction of GFP^+^ cells, indicating that MND1 indeed facilitates HR in somatic cells (Fig. 2F). Consistent with the notion that MND1 may facilitate some, but not all forms of HR‐dependent repair, loss of MND1 led to a partial reduction in HR frequency, far less than the reduction obtained by depletion of RAD51. Reduction of HR efficiency in the DR‐GFP assay is rescued by ectopic expression of MND1 in a setting where endogenous MND1 is depleted using an siRNA that targets the 3′UTR of MND1, which is not present in the rescue construct (Fig. 2G,H).

Homologous recombination in somatic cells is largely limited to S and G2 phase [12, 59], and therefore, the reduction in HR observed after depletion of MND1 could be induced indirectly, through an altered cell cycle distribution in MND1‐deficient cells. However, we show that the cell cycle distribution of U2OS cells depleted of MND1 is comparable to control cells (Fig. S2K), excluding cell cycle differences as a cause for the decrease in GFP^+^ cells. Thus, we conclude that MND1 plays an important and direct role in the repair of DSBs via HR in somatic cells.

After we defined a novel role for MND1 in somatic HR, we were interested to further understand which repair pathway is taking over after MND1 loss. We have already seen a mild sensitization of ΔMND1 cells toward DNAPKi, indicating that these cells come to rely more on NHEJ for repair (Fig. S2I). To extend our knowledge on pathway choice, we investigated the SSA repair pathway usage after MND1 loss. SSA is a highly mutagenic repair pathway that involves long‐range DNA resection. When testing a previously published SSA reporter cell line in U2OS background (SA‐GFP), we found a slight increase, but no significant change in SSA usage after MND1 depletion (Fig. 2I). Therefore, we conclude that there is no increase in SSA repair after MND1 loss.

To further assess not only NHEJ but also MMEJ usage in ΔMND1 cells, we analyzed recovery after IR in RPE1 CCB1‐YFP cells, where mitotic entry of G2 phase cells can be easily assessed in live cells. In unirradiated WT or ΔMND1 cells, mitotic entry is not perturbed by inhibition of either NHEJ (by DNAPKi) or MMEJ (by POLQi; Fig. S2L). When treated with a low dose of irradiation (0.5 Gy), WT G2 cells depend on DNAPKc‐mediated NHEJ for repair, as treatment with a DNAPKi reduces mitotic entry (Fig. 2J). In contrast, inhibition of MMEJ by a POLQi does not affect mitotic entry in WT G2 cells, indicating no involvement of POLQ‐mediated MMEJ in WT G2 phase cells. This changes dramatically in ΔMND1 cells. In cells lacking MND1, mitotic entry after IR is markedly reduced, and inhibition of either NHEJ or MMEJ causes an even further reduction in mitotic entry, which indicates that both MMEJ and NHEJ are required for ΔMND1 cells to repair DSBs in G2 phase. Furthermore, inhibition of both NHEJ and MMEJ together in ΔMND1 cells leads to an additional decrease in mitotic entry. Therefore, we conclude that in ΔMND1 cells, compensation of the repair defect largely occurs via MMEJ‐mediated DSB repair.

### 3.3 Loss of MND1 leads to increased sensitivity to some, but not all types of DSBs and specifically impairs HR in G2 phase

**Fig. 3:**
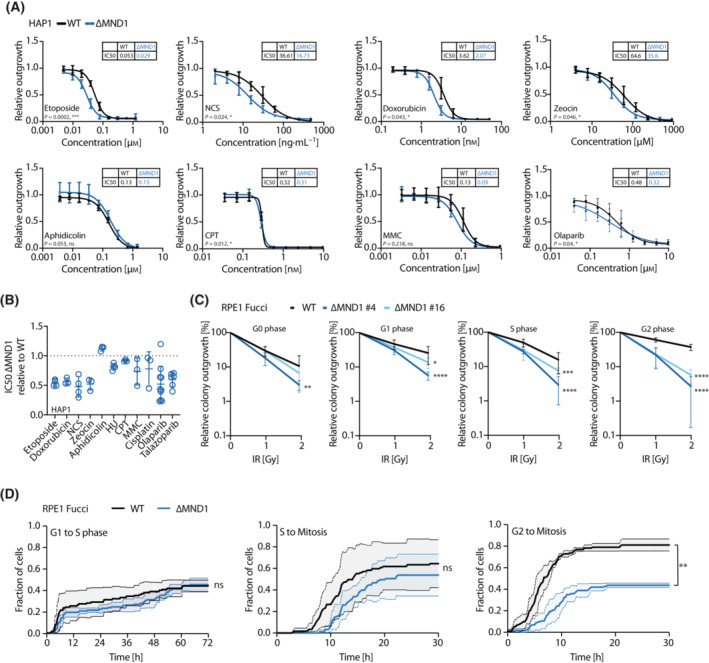
MND1 loss sensitizes to induction of a specific species of DSBs. (A) Drug‐response assays with etoposide (*N* = 4), neocarzinostatin (NCS, *N* = 4), doxorubicin (*N* = 3), zeocin (*N* = 3), aphidicolin (*N* = 3), camptothecin (CPT, *N* = 3), mitomycin C (MMC, *N* = 3), olaparib (*N* = 10), and talazoparib (*N* = 6) in HAP1 WT and ΔMND1 cells. Data presented as mean ± SD. Statistical analysis is performed on IC50 values (Fig. S3B). (B) Relative IC50 values of drug‐response curves from Fig. 2A and Fig. S2A. IC50 values of ΔMND1 HAP1 cells are normalized to IC50 values of WT cells. Each dot represents a replicate experiment. (C) Clonogenic outgrowth of RPE1 iCut Fucci WT and two ΔMND1 clonal cell lines after treatment with IR and sorting into different gates. Data presented as mean ± SEM, *N* = 6. Statistics depict *P*‐values (*P ≤ 0.05, **P ≤ 0.01, ***P ≤ 0.005, ****P ≤ 0.001) after analysis using a Fisher's test. (D) Analysis of cell cycle progression of RPE1 Fucci WT and ΔMND1 cells. G1 phase cells were followed into S phase (left), and S and G2 phase cells were followed into Mitosis (middle and right, respectively).

Homologous recombination is an important repair mechanism at sites of endogenously induced DSBs. Ongoing replication is the source of the majority of endogenously occurring DNA lesions, most of which are repaired through HR. After establishing the role of MND1 in somatic HR after IR (Fig. 2D), we aimed to assess whether this can be extended to other sources of DSBs, like replication‐associated damage. We find significantly increased sensitivity toward the different DSB‐inducers etoposide and doxorubicin (both topoisomerase II inhibitors), as well as the radiomimetic drugs neocarzinostatin (NCS) and zeocin (Fig. 3A,B and Fig. S3B). By contrast, when we treated HAP1 ΔMND1 cells with inducers of replication stress, hydroxyurea (HU) and aphidicolin, there was little to no difference in the sensitivity of the MND1‐deficient cells as compared to their wild‐type counterparts (Fig. 3A,B and Fig. S3A,B). Similar to replication stress induction, MND1 loss did not sensitize cells to camptothecin (CPT; Fig. 3A and Fig. S3B), a topoisomerase I inhibitor that creates DSBs specifically in S phase, when a replication fork collides with the blocked topoisomerase I complex [60]. These data imply that MND1 is not required for the repair of DSBs that arise during DNA replication. To corroborate this finding, we also tested whether MND1 is involved in the repair of intrastrand DNA crosslinks (ICL), which often result in DSBs during replication [61, 62]. We find no sensitization of ΔMND1 cells toward the ICL‐inducing drugs cisplatin and mitomycin C (Fig. 3A,B and Fig. S3A,B). This shows that MND1 is not involved in the HR‐dependent repair of ICLs. At last, we treated cells with olaparib and talazoparib, two PARP inhibitors (PARPi) commonly used in the clinic. Comparing to data obtained in BRCA1/2‐deficient cells, which are exquisitely sensitive to PARPi treatment [6, 63], we also observe a moderate sensitization toward olaparib and talazoparib after MND1 loss (Fig. 3A,B and Fig. S3A,B). As PARPi treatment introduces PARP‐trapping lesions that are converted to DSBs during S phase [63], this indicates that there is some involvement of MND1 in the repair of replication‐associated DSBs. However, we cannot exclude that this could be selectivity between DNA break structures having differential requirements for MND1. Taken together, these data show that the requirement of MND1 for the response of cells toward replication stress is limited to the highly HR‐dependent PARPi‐induced breaks.

We were intrigued by the differential requirement for MND1 in response to DNA damage that is or is not associated with replication. We reasoned that this difference could be because either (a) the MND1‐HOP2 complex is inhibited during S phase and therefore not involved in the response to replication‐associated breaks, (b) HR in S phase can in large part occur via strand exchange, rather than strand invasion, which can occur independent of MND1, or (c) differences in the repair of one‐ and two‐ended DSBs that determine whether the MND1‐HOP2 complex is necessary. To address the importance of MND1‐HOP2 complex in various cell cycle stages after IR, we assessed the clonogenic outgrowth of RPE1 Fucci cells sorted from distinct cell cycle phases (cycling G1, S, and G2 phase cells (Fig. S3C,D)). We observed significant sensitization in most ΔMND1 conditions compared with WT, with the exception of ΔMND1 clone 16 in G0 phase (Fig. 3C). Strikingly, the further cells progressed through the cell cycle, the stronger the sensitization after MND1 loss became. We observed mild sensitization after MND1 loss in G1 and S phase cells. Notably, sensitization was much more prominent in G2 phase (Fig. 3C), consistent with our finding that depletion of MND1 affects repair in G2 cells only (Fig. 2C). Interestingly, we observe no defect in DSB resolution in S phase (Fig. 2C), whereas we observe a sensitization of S phase cells to IR in our clonogenic outgrowth (Fig. 3C). As the observed sensitization in early cell cycle phases can be caused by carry‐over of DSBs into G2, we decided to study the RPE1 Fucci cells at the single‐cell level and follow individual cells through the cell cycle. When following irradiated G1 phase cells into S phase and S phase cells into Mitosis, we could not observe a significant difference between WT and ΔMND1 cells (Fig. 3D and Fig. S3E). Only cells irradiated in G2 phase showed a significant sensitization after MND1 loss when followed into Mitosis. This implies that the sensitization of MND1 cells that we observe after IR is due to a repair defect in G2, which is also affecting G1 and S phase cells that carry over the damage into G2 phase.

Taken together, we assume that a carry‐over of DSBs from G1 and S phase into G2 is the most likely explanation of our observation in our Fucci cell sorting, as live‐cell analysis of single cells shows no difference between WT and ΔMND1 cells in G1 and S phase. Thus, our data show that the role of MND1 in DNA repair is mostly restricted to G2 phase cells and the repair of two‐ended DSBs.

### 3.4 MND1 forms foci at DNA DSB sites together with yH2AX and RAD51

**Fig. 4:**
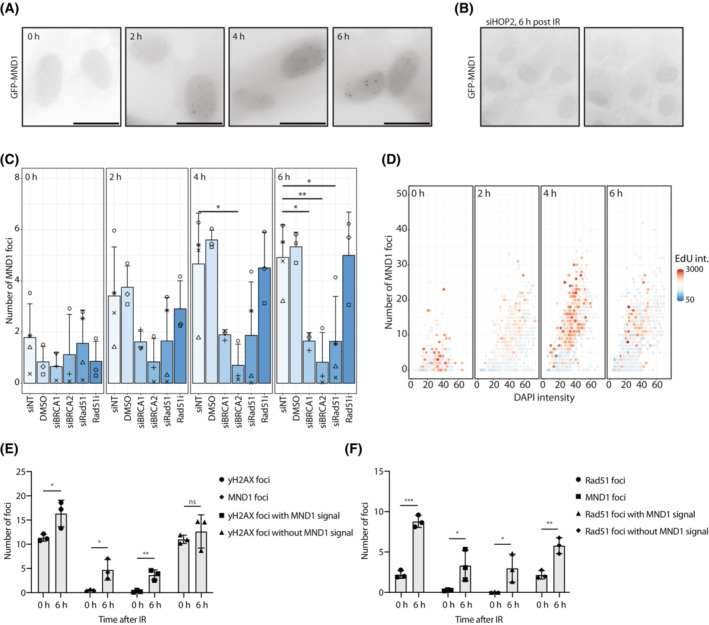
Localization of MND1 to a subset of yH2AX and RAD51‐coated DSB locations is dependent on resection. (A) Example images of RPE1 HALO‐53BP1_RPA1‐mScarlet2_GFP‐MND1 cells after 4 Gy of IR (Quantification in C, *N* = 3). Depicted are GFP‐MND1 imaging examples at 0, 2, 4, and 6 h after 4 Gy. Scale bar: 20 μm. (B) Example images of cells from (A) treated with siHOP2 for 24 h before IR with 4 Gy. Images are taken 6‐h post‐IR (*N* = 3). (C) Number of GFP‐MND1 foci detected at 0, 2, 4, and 6 h after 4 Gy IR. RPE1 HALO‐53BP1_RPA1‐mScarlet2_GFP‐MND1 cells were treated with different siRNAs/inhibitors for HR pathway proteins. Displayed are three independent replicates (mean ± SD). Statistical analysis (*t*‐test) was performed on mean values (*P ≤ 0.05, **P ≤ 0.01). Example images are displayed in (A). (D) Co‐staining of GFP‐MND1 with DAPI and EdU to indicate the cell cycle phase‐dependent formation of GFP‐MND1 foci. These data are a representative experiment of two replicate experiments. (E) Overlap of GFP‐MND1 and yH2AX foci. Depicted are the number of either total yH2AX or MND1 foci, yH2AX foci with(out) MND1 signal, or MND1 foci with(out) yH2AX signal. Displayed are three independent replicates (mean ± SD). (F) Overlap of GFP‐MND1 and RAD51 foci. Depicted are the number of either total RAD51 or MND1 foci, RAD51 foci with(out) MND1 signal, or MND1 foci with(out) RAD51 signal. Displayed are three independent replicates (mean ± SD). Statistical analysis of (E, F) was performed using unpaired *t*‐test (*P ≤ 0.05, **P ≤ 0.01, ***P ≤ 0.005).

So far, we established that MND1 facilitates the HR repair of two‐ended DNA DSBs during the mitotic cell cycle. However, we have not yet investigated whether MND1 localizes to DNA DSBs and is therefore directly involved in the repair of DSBs. For this, we visualized MND1 localization to sites of DNA damage by exogenous expression of a GFP‐tagged MND1 fusion protein (GFP‐MND1) in the RPE1 HALO‐53BP1 cell line (Fig. S4A). We have previously demonstrated that this GFP‐tagged MND1 construct rescues sensitivity in HAP1 cells to a similar extent as untagged MND1 (Fig. 1E), warranting us to use this construct for further imaging experiments. GFP‐MND1 readily forms foci after DNA damage induction as early as 2 h after IR, and foci numbers continue to increase until they reach a plateau between 4 h and 6 h after DNA damage induction (Fig. 4A, quantification of different conditions in Fig. 4C). Since MND1 and HOP2 function epistatically (Fig. 1E), we tested whether MND1 foci formation depends on HOP2. Interestingly, we see no foci formation after HOP2 depletion by siRNAs (Fig. 4B and Fig. S4B), showing that MND1 foci formation is entirely dependent on its co‐factor HOP2. These data show that MND1 is recruited to sites of DSBs and that MND1 recruitment requires HOP2.

To further elucidate the notion that MND1 foci formation is dependent on resection of the DSB and ongoing HR repair, we depleted canonical HR factors using siRNAs. We find that MND1 recruitment is fully dependent on BRCA1, BRCA2, and RAD51 (Fig. 4C). This places MND1 localization to sites of DSBs downstream of DNA end‐resection and RAD51‐loading, which is consistent with MND1's role during meiosis [26]. We also tested whether the chemical inhibition of RAD51 would affect MND1 foci formation, using an inhibitor that specifically blocks sister chromatid exchange, but does not prevent RAD51 from binding to ssDNA [19]. Interestingly, we do still observe MND1 foci formation in this setting, which indicates that it is the presence of RAD51 at sites of ssDNA that is important for MND1 recruitment, whereas its functionality is dispensable. Therefore, we conclude that MND1 recruitment to sites of damage is dependent on the presence of RAD51‐coated ssDNA and occurs prior to RAD51‐dependent invasion of the sister chromatid.

Consistent with the role of MND1 in the response to IR‐induced DSBs that is primarily restricted to repair in G2 (Fig. 3C), we find that MND1 is recruited to foci in S and G2 phase (Fig. 4D). Conversely, MND1 foci are not present in G1 phase. This indicates that MND1 is either involved in both S and G2 phase at sites of damage, or the loading of MND1 during S phase is a preparation for when it is required later in G2.

Next, we aimed to analyze whether MND1 foci are formed at sites of DNA damage, and more specifically, sites of HR repair. We find that virtually all GFP‐MND1 foci are positive for yH2AX and RAD51 (Fig. 4E,F and Fig. S4D), demonstrating that MND1 exclusively localizes to sites of active HR repair. This is also observed when staining for foci formation of endogenous HOP2 protein (Fig. S4G), where we observe foci formation at sites of yH2AX phosphorylation. These HOP2 foci furthermore increase in number during the course of the cell cycle, similar to what we observed for GFP‐MND1 foci previously in Fig. 4D. However, only a fraction of IR‐induced yH2AX‐ or RAD51 foci are coated with GFP‐MND1, consistent with the fact that not all DSBs engage in HR and only a subset of HR breaks requires MND1 for their resolution (Fig. 4E,F). This again indicates that, of the breaks that engage in RAD51‐dependent HR, there is only a subset that depends on MND1 for repair.

Considering that MND1‐HOP2 has a known role at telomeres during ALT [29], we aimed to exclude that the GFP‐MND1 foci we observe are only formed at telomeric DSBs at telomeres. For this, we used CRISPR/Cas9 to generate DSBs either at telomeres (‘Telo’) or in GAPDH pseudogenes (‘P63’, this crRNA generates around 18 DSBs in RPE1 cells in nontelomeric regions [35]). When we induced DSBs at these distinct locations, we could observe that even though the overall induction of DSBs is higher when using the crRNA targeting telomeres (as shown by quantification of 53BP1 foci), the number of GFP‐MND1 foci is smaller (Fig. S4E). This shows that the observed recruitment of GFP‐MND1 to foci is not restricted to DSBs induced at telomeres and strengthens our data showing a general role of MND1 in the HR repair of DSBs.

The treatment of cells with ionizing irradiation is well known to induce a broad range of DNA damage types, both DSBs and SSBs. To delineate whether GFP‐MND1 foci form exclusively at sites of DSBs, we treated cells with SSB inducers aphidicolin (Aph), hydroxyurea (HU), and ultraviolet (UV) light. When assessing the generation of GFP‐MND1 foci in comparison with IR, we observe that MND1 foci are formed after IR treatment (Fig. S4F), while no MND1 foci are observed after treatment with Aph, HU, or UV even though the level of yH2AX is comparable, and even higher after UV treatment. These data support our hypothesis that MND1 is acting mainly at sites of DSBs.

### 3.5 MND1‐deficient cells are prone to arrest at the G2 checkpoint after DSB induction

**Fig. 5:**
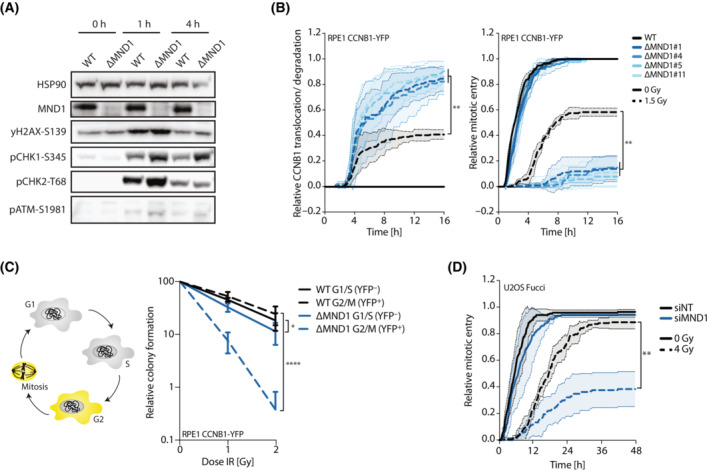
Loss of MND1 leads to hyperactivation of the G2 checkpoint and cell cycle arrest. (A) Immunoblotting of checkpoint activation in RPE1 CCNB1‐YFP WT and ΔMND1 cells. Blotting was performed at 0, 1, or 4 h after 4 Gy of IR (*N* = 2). (B) RPE1 CCNB1‐YFP cells were irradiated with 1.5 Gy and CCNB1 translocation (left) or mitotic entry (right) is plotted over time in either WT or different ΔMND1 cell lines. Control cells that were not irradiated are depicted in full line. Data presented as mean ± SD (*N* = 3). Statistical analysis (*t*‐test) is performed at 16 h between WT and the separate ΔMND1 clones (**P ≤ 0.01). (C) Colony outgrowth was assessed in RPE1 CCNB1‐YFP WT and ΔMND1 cells after increasing doses of IR. Cells were sorted into YFP^−^ (G1/S) and YFP^+^ (G2) cells after IR. Data are presented as mean ± SD (*N* = 3). Statistical analysis was performed using a Fisher's test (*P ≤ 0.05, ****P ≤ 0.0001). (D) Relative mitotic entry of G2 cells after 4 Gy of IR. U2OS Fucci cells were treated with NT, MND1, and HOP2 targeting siRNAs and G2 cells were followed until mitotic entry. Data presented as mean ± SD (*N* = 3). Statistical analysis (*t*‐test) is performed at 48 h (**P ≤ 0.01, ***P ≤ 0.001, ****P ≤ 0.0001).

Thus far, we established that MND1 plays an important role in the repair of DSBs by aiding HR and that loss of MND1 leads to increased DSB toxicity. It has been previously established that defects in DSB repair lead to stronger activation of cell cycle checkpoints via ATM and ATR, explaining growth arrest in those cells [3, 64, 65]. Specifically, it has been shown that HR defects lead to increased ATR‐dependent cell cycle exit [66]. Thus, we were wondering whether the HR defects we observe after MND1 loss (Fig. 2C–E) lead to increased cell cycle checkpoint activation. Indeed, we see a persistent increase in general DNA damage signaling in RPE1 ΔMND1 cells when assessing phosphorylation of H2AX (S139, yH2AX), CHK1 (S345), and CHK2 (T68), which demonstrates hyperactivation of ATM‐ and ATR‐dependent checkpoint signaling upon loss of MND1 (Fig. 5A). Consistently, we also observe a persistent increase in pCHK1‐S345 in HAP1 ΔMND1 cells (Fig. S5A). Quantification however indicates that the effect of MND1 loss on ATM activation (pATM and pCHK2 signaling) is variable (Fig. S5B). To the contrary, ATR hyperactivation in ΔMND1 cells is consistent not only between experiments but also between cell lines. Therefore, we conclude that MND1 loss leads to exacerbated ATR activation, while more experiments will have to be performed to resolve if the unprocessed DSBs can also trigger sustained ATM activation. However, we see clear evidence for the presence of resected ssDNA present [3], which shows that loss of MND1 is leading to increased checkpoint signaling which is potentially causing the growth arrest of ΔMND1 cells in response to DSB induction.

We have previously shown that HR intermediates can trigger a permanent cell cycle exit in G2 phase [66]. This is because unresolved RPA and RAD51‐coated DSBs elicit a strong G2 checkpoint response which leads to nuclear translocation or degradation of cyclin B1, a marker of irreversible cell cycle arrest [67, 68]. We reasoned that HR intermediates that are left unresolved in ΔMND1 cells are causing a strong arrest specifically in G2 phase to prevent cells from entering mitosis. To test whether MND1 loss induces a G2 arrest, we made use of an RPE1 cell line with endogenously tagged cyclin B1 (RPE1 CCNB1‐YFP; [68, 69]), where we knocked out MND1 (Fig. S5C). Indeed, cells deficient for MND1 show a dramatic increase in nuclear translocation or degradation of cyclin B1 and a corresponding decrease in mitotic entry (Fig. 5B). This is also evident when we deplete MND1, and its co‐factor HOP2, by siRNA treatment (Fig. S5D). This demonstrates that the loss of MND1 potentiates the arrest and permanent cell cycle withdrawal by IR in G2 phase.

As we have now seen MND1 loss to infer a dramatic reduction in mitotic entry, co‐occurring with an exacerbated G2 checkpoint activation, we tested whether G2 cells in this system are more sensitive toward IR than G1 cells. For this, we irradiated WT and ΔMND1 RPE CCNB1‐YFP cells and then sorted both a YFP^−^ and YFP^+^ population for clonogenic outgrowth (G1 and G2 phase cells, respectively, Fig. S5E). We indeed find that the G2 (YFP^+^) ΔMND1 cells are specifically sensitive toward IR when compared to WT cells (Fig. 5C). These data are consistent with a role for MND1 in HR during G2 phase and its loss resulting in defects in strand invasion, producing persistent HR intermediates that can drive a permanent cell cycle arrest after DNA damage.

Lastly, we tested whether the loss of MND1 also limited the proliferation of transformed cells that receive DSBs in G2. For this, we imaged U2OS Fucci cells that were depleted of MND1 using siRNAs. When we followed single G2 phase cells entering mitosis, we confirmed that loss of MND1 reduces mitotic entry specifically upon IR (Fig. 5D). We conclude that loss of MND1 results in a stronger G2 checkpoint, thereby increasing radiation sensitivity.

## 4 Discussion

Here, we identified for the first time a previously unrecognized role for the MND1‐HOP2 complex during somatic HR in G2 phase. We show that cells lacking MND1 demonstrate increased sensitivity toward DNA damage, specifically y‐irradiation and drug‐induced two‐ended DSBs. Similar to meiosis, where MND1 and HOP2 bind RAD51 and facilitate strand invasion and D‐loop formation [28, 58], we demonstrate the importance of MND1 for efficient HR in somatic cells. Furthermore, complex formation of MND1‐HOP2 is necessary for the localization of MND1 to sites of (RAD51‐covered) DSBs, where it facilitates repair.

When we depleted MND1 in multiple cell lines, we found that all but one of the tested cell lines were sensitized to IR upon MND1 loss (Fig. S1I). We have yet to identify the underlying reason for this difference between cell lines. One possible explanation is that, since the MND1‐HOP2 complex is specifically active in G2 phase cells, cell insensitive to MND1 loss can largely execute repair outside of G2. However, we find that the percentage of G2 phase cells does not correlate with the sensitization of cells after MND1 loss. The difference in effect is also not explainable by mutations present in MND1 or HOP2 in the tested cell lines, as sequence analysis confirms the presence of WT products (data not shown). Therefore, we can only speculate what the underlying reason for these differences could be. One possibility is that different cell lines have varying dependencies on HR during G2 phase, where we see MND1/HOP2 to be active. To test this, further analysis of HR preference of these different cell lines has to be undertaken.

MND1 was not previously identified having a general role in repair in somatic cells, and we hypothesize that previous studies performed in asynchronous cells have masked this cell cycle phase‐specific role of MND1. This leads us to speculate that there are still other genes with unidentified roles in the DDR with such a specific involvement in DNA repair.

The formation of GFP‐MND1 foci upon IR (Fig. 4) establishes the direct involvement of MND1 at sites of damage. We confirmed that the recruitment of MND1 to DSBs is entirely dependent on HOP2 and the initiation of HR, up to successful RAD51 loading (Fig. 4B,C). This is in line with previous experiments performed *in vitro*, where MND1 binds to established RAD51‐coated ssDNA, to thereby aid in D‐loop formation [26, 28, 58]. The observed localization of GFP‐MND1 to sites of DSBs is in contrast to previous studies, where in yeast meiotic cells MND1 foci were found to be formed randomly throughout the nucleus, rather than at sites of RAD51 foci [48, 50]. The difference between our observation and previously published data could be underlying differences in MND1 usage between yeast and mammalian cells as MND1 and HOP2 are only in mammalian cells expressed during both the meiotic and the mitotic cell cycle. Therefore, we propose that there are critical differences in the function of MND1 in its localization to sites of DSBs between yeast and mammalian systems, and it would be interesting to study whether MND1 is recruited to RAD51‐covered DSBs in mammalian meiotic cells. Due to a lack of suitable antibodies for immunofluorescence staining, we limited our study on localization of MND1 to DSBs to the imaging of the ectopic overexpression of a GFP‐tagged MND1. We have shown in Fig. 1E that the expression of GFP‐MND1 rescues the IR sensitization to the same level as untagged MND1 overexpression and in Fig. 2F that GFP‐MND1 overexpression rescues RAD51 foci resolution in a ΔMND1 cell line. Furthermore, we can observe foci formation of endogenous HOP2 protein in cells that do not overexpress MND1, which implies that recruitment of the HOP2/MND1 complex is not an artifact of MND1 overexpression (Fig. S4E,G). Therefore, we are confident that our imaging studies represent the behavior of endogenous MND1 protein.

We show that during somatic DSB repair, MND1 is readily recruited to DSBs covered with both yH2AX and RAD51 (Fig. 4E,F). However, not all yH2AX or RAD51 foci recruit MND1 (Fig. 4E,F). This goes together with our data showing that (a) MND1 loss is less detrimental to 53BP1 foci resolution than depletion of BRCA1 (Fig. 2A) and (b) the HR efficiency in the DR‐GFP assay is reduced to ~ 50%, whereas RAD51 depletion leads to full loss of HR. This indicates that MND1 is required for repair of only a subset of DSBs engaged in HR. However, it is possible that we do not observe full overlap of RAD51 foci with MND1 as these experiments are not stratified for cell cycle. We hypothesize that MND1 foci in S phase localize less to RAD51 than in G2 phase, because even though MND1 can localize to foci in S phase, its role in repair during S phase is minor (foci resolution is unchanged and S phase cells are less radiosensitive than G2 cells, Figs 2B and 3C). Further experiments have to be conducted to establish the role of MND1 in S phase compared with G2 phase and to identify a determining factor for the conditions and types of breaks that require the MND1‐HOP2 complex for their repair.

When analyzing the response of ΔMND1 cells toward different types of damaging agents, we observed a striking difference between induction of classical DSBs and SSB inducers that convert into DSBs during replication. This is particularly interesting in the context of the dramatic sensitization to IR that we observe. Ionizing irradiation does not only induce DSBs but also a plethora of other types of damage like SSBs and base damage [9]. Therefore, it is interesting to note that when induced separately, SSBs do not seem to induce increased sensitivity in ΔMND1 compared with WT cells. These SSBs however were introduced using various chemicals, which are not identical to the SSBs induced by IR. The exact effect of other types of lesions by IR outside DSBs is therefore not addressed in this study.

We have uncovered that not all HR‐mediated repair requires MND1, which leads to speculate what the differences of these DSBs are. Based on our observations, we hypothesize that MND1 is required for repair of only two‐ended DSBs, and not one‐ended DSBs. Strikingly, the largest sensitization to IR upon MND1 loss was observed when DSBs were induced in G2, outside the context of replication. Moreover, when DSBs were generated by replication stress inducers aphidicolin, CPT or HU, only a slightly increased sensitization could be observed after HU treatment only (Fig. 3A,B). During meiotic recombination, the homologous chromosomes used for crossovers are already compacted and apart from each other, calling for the requirement of efficient search of the homologous region. This has been reported to require the presence of the MND1‐HOP2 complex [70]. We propose that our observed S phase sensitization to IR is carry‐over of DSBs from G1/S phase into G2, where the role of MND1 is the most prominent. This is supported by our live‐cell imaging experiment, where we followed single RPE1 Fucci cells (Fig. 3D). We speculate that this specificity reflects a unique role of the MND1‐HOP2 complex in the repair of DNA damage, where replication has concluded and the DNA strands are closed and sister chromatids taken apart, similar to the role of the MND1‐HOP2 complex in meiosis. Therefore, more complex methods for homology search are necessary, spanning longer distances in the nucleus. We envision that DSBs after successful replication without the sister chromatid in direct proximity require an advanced mechanism for homology search and strand invasion. The lack of sensitization of ΔMND1 cells toward classical replication stress inducers supports this model. It should however be noted that we do also observe MND1 foci in S phase (Fig. 4D), but no apparent requirement for MND1 in foci resolution at this stage. We hypothesize that MND1 is normally recruited to RAD51‐coated stretches of DNA in S phase, but its contribution is nonessential to complete replication‐associated HR, which is the reason why we only observe a mild repair defect at this stage. This could be a consequence of strand exchange‐driven HR, which may well have different requirements than stand invasion‐driven HR. Furthermore, both MND1 and HOP2 are nonessential in human somatic cells. This is in striking contrast to other, ‘classical’, HR factors like RAD51, BRCA1, and BRCA2, which are involved in the repair of replication‐associated DSBs as well [20, 71, 72], are essential factors for cell survival.

What makes this specific role of the MND1‐HOP2 complex so interesting is that these proteins are by themselves dispensable for normal cell survival. As MND1‐HOP2 are not involved in the replication‐associated repair of DSBs, and consequently not involved in the repair of most endogenous DNA damage, their loss is dispensable for cellular survival under normal growth conditions. However, their loss renders cells highly sensitive to induction of exogenous damage by, for example, IR or TOP2i (as seen in Figs 1 and 3). Therefore, the interference with MND1‐HOP2 function in cells could be a potentially interesting approach for cancer combination treatment with targeted DSB induction. This has the potential to be an efficient treatment, specifically in G2‐phase‐rich tumors.

## 5 Conclusion

We find MND1 as a crucial player in the HR repair of DNA DSBs, specifically during G2 phase of the cell cycle. This specificity for replication‐independent repair of DSBs could open new possibilities for therapeutic intervention, specifically in HR‐proficient tumors.

## Author contributions

LKo, LKr, and RHM conceived and designed the study. LKo, AF, LB, ESK, EB, and MS performed experiments, data processing, and figure preparation. BB wrote the Fiji plugin for image processing and analysis. The screen was performed and analyzed by FMF and VAB under the supervision of TRB. LKo, LKr, and RHM wrote the manuscript with input from all authors.

## Conflict of interest

The authors declare no conflict of interest.

## Supporting information


**Fig. S1.** Confirmation of IR sensitivity phenotype.
**Fig. S2.** MND1 is involved in somatic HR.
**Fig. S3.** Loss of the MND1‐HOP2 complex sensitizes cells toward DSB induction.
**Fig. S4.** Foci formation of GFP‐MND1 in RPE1 cells.
**Fig. S5.** Checkpoint activation in MND1 deficient cells.
**Table S1.** IR screen results.

## Data Availability

Raw data of the IR screen can be found in Supporting Information.
